# Artificial Intelligence Can Inform Prediction of American Society of Anesthesiologists Physical Status Classification in Hand Surgery

**DOI:** 10.1177/15589447251383047

**Published:** 2025-10-29

**Authors:** Thompson Zhuang, Margaret Bohr, Nathaniel Disser, Julio Ojea Quintana, Ethan Liu, Andrew D. Sobel, David Bozentka, Ines C. Lin, Hannah H. Lee

**Affiliations:** 1 Hospital of the University of Pennsylvania, Philadelphia, USA

**Keywords:** artificial intelligence, ChatGPT, generative AI, risk prediction, safety

## Abstract

**Background:**

Hand surgeons are increasingly asked to assign American Society of Anesthesiologists (ASA) physical status classifications for local anesthesia–only procedures despite minimal training. Artificial intelligence (AI) is a powerful tool that could assist in ASA class assignment. We evaluated the concordance/agreement between surgeon-rated and AI-rated ASA class.

**Methods:**

Local anesthesia–only hand surgical episodes (n = 236) performed by 4 fellowship-trained hand surgeons at ambulatory surgery centers were included. We used ChatGPT to estimate ASA class–based patient medical history. Interrater agreement between surgeon and AI ratings were compared with the weighted Cohen κ. The most recent anesthesia-rated ASA class from another anesthesia event was also recorded (if present) and compared. We used multivariable logistic regression models to evaluate whether patient factors were associated with concordance. We also recorded 30-day postoperative emergency department visits and hospitalizations.

**Results:**

Overall concordance between surgeon and AI ratings was 56%. In 72% of discordant cases, surgeon-rated ASA class was lower than the AI rating. Surgeon-rated ASA class exhibited fair agreement with the AI rating (κ = 0.38). Artificial intelligence–rated ASA class exhibited moderate agreement with prior anesthesia-rated ASA class (κ = 0.44). In the multivariable model, age, sex, and presence of a prior anesthesia-rated ASA class were not associated with concordance between surgeon and AI ratings. Postoperative emergency department visits or hospitalization occurred in 7 (3%) patients, for which surgeon-rated ASA class was lower than AI rating in 4 cases.

**Conclusions:**

Generative AI could be used as a tool to support ASA class assignment for local anesthesia–only hand surgeries.

## Introduction

Hand surgery is increasingly and predominantly performed in the outpatient and procedure room settings with the patient wide awake under local anesthesia only without anesthesiologist involvement,^1,2^ which offers improved operating room efficiency and lower cost.^3^ In these cases, hand surgeons often assume responsibility for patient risk stratification in the form of assigning an American Society of Anesthesiologists (ASA) Physical Status classification when required by institutional protocols.^4^ These protocols exist to identify high-risk patients who may benefit from enhanced preoperative workup and/or anesthesia-trained provider involvement (eg, monitored anesthesia care). Very high-risk patients may not be candidates for procedures in freestanding surgery centers. The ASA classification is a system for predicting perioperative risk (eg, medical complications, readmission, and mortality) that ranges from class I to VI, in which a higher ASA class denotes an increased severity of comorbid systemic disease.^5^ The ASA classification has been validated across a broad range of surgical procedures as a predictive factor for the risk of complications,^5,6^ including in orthopedic surgery.^7-9^ In hand surgery, higher ASA class has also been associated with a higher risk of postoperative complications such as surgical site infection and delayed recovery.^10,11^

Despite the widespread utilization of the ASA classification for perioperative risk stratification, surgeons receive little formal training on assigning ASA class compared with anesthesiologists. Previous studies have shown high discordance rates between ASA class assigned by surgeons as compared with anesthesiologists,^4,12^ which could lead to inaccurate perioperative counseling and performance of surgery in an inappropriate operative setting (eg, freestanding vs hospital-based surgery center). Thus, a tool to assist surgeons in ASA class assignment would be beneficial. The advent of artificial intelligence (AI) offers a powerful tool for standardized, automated risk stratification that could be applied for ASA classification and readily integrated into clinical workflows,^13^ thereby alleviating the burden on hand surgeons who routinely perform cases under local anesthesia without an anesthesia provider. In addition, high concordance between AI and anesthesiologist ASA classifications has been shown, suggesting that the use of AI could help align surgeon ASA class assignment with that of an anesthesiologist.^14^ However, the potential role of AI in ASA class assignment in hand surgery has not been explored. In this study, we evaluated the concordance or interrater agreement between surgeon-rated and AI-rated ASA class to identify discrepancies that might be improved by the use of AI.

## Methods

### Data Collection

This study was approved by our institutional review board. All local anesthesia–only hand ambulatory surgical episodes (n = 264) from 4 hand surgeons at a single institution from March 2024 through January 2025 were retrospectively captured. Cases were captured retrospectively to ensure consistency in the AI model and data entry. All cases were performed at ambulatory surgery centers that required formal surgeon-documented ASA class assignments, which began in January 2024. Surgeon years in practice varied from 4 to 34 years, including 1 plastics-trained and 3 orthopedics-trained surgeons with subspecialty certificates in surgery of the hand. Patient demographic information, procedure including laterality, date of surgery, surgeon, and the incidence of and reasons for emergency department (ED) visits or hospitalizations within 30 days after surgery were recorded. Each procedure had an ASA Physical Status classification assigned by the surgeon on the date of surgery, except for 28 cases (11%) that were excluded from the analysis. Overall, 236 discrete surgical episodes were evaluated, consisting of 278 individual procedures (Table 1). The hand surgeons received no specific training in assigning the ASA Physical Status classification, and no uniform checklist or set of criteria were distributed. The most recent prior ASA classification that was performed by an anesthesiologist (eg, for a preceding procedure) was also recorded, if present. This prior ASA class was present for 164 (69%) episodes. The average time between the prior anesthesia ASA rating and date of surgery was 738 (SD = 858) days.

**Table 1.** Procedure Mix.

Procedure	Frequency, n
Trigger finger release	121
Carpal tunnel release	75
Cyst or mass excision	46
First dorsal compartment release	16
Other	
Biopsy	6
Bone/osteophyte excision	5
Removal of hardware	3
Foreign body removal	2
Flexor/extensor tenolysis	2
Tendon repair	1
Manipulation under anesthesia	1

We used ChatGPT (GPT-4o; OpenAI, San Francisco, California), a generative AI model, to predict each patient’s ASA class. The following standardized script was entered into the ChatGPT interface: “Estimate ASA class based on the following past medical history: . . .” To simulate the fast-paced clinical environment in which this model would be deployed in practice, the patient’s past medical history as given in the electronic medical record (EMR) was directly pasted into the script above, without patient identifiers. If there was no past medical history, the patient’s active problem list was used. No other information was provided to the AI model. In 36% (n = 85) of cases, the ChatGPT output contained 2 possible ASA classes, dependent on a clinical assessment of patient status. In the primary analysis, only the lower of the 2 possible classes was used. In a sensitivity analysis, we used the higher ASA class, when present, to evaluate whether this would alter the result. Entries into the ChatGPT model were performed between August 2024 and March 2025.

### Statistical Analysis

A simple concordance metric was measured as the proportion of cases in which ASA rating pairs (eg, surgeon and AI ratings) were equal. In cases where the surgeon and AI ratings were discordant, we measured whether the surgeon rating was greater or less than the AI rating. Interrater agreement was reported using a quadratically weighted Cohen κ, which penalizes larger deviations between pairs. Generally, κ coefficients of 0 to 0.2 denote poor agreement, 0.2 to 0.4 denote fair agreement, 0.4 to 0.6 denote moderate agreement, 0.6 to 0.8 denote substantial agreement, and 0.8 to 1.0 denote almost perfect agreement.^15^ We performed an additional analysis in which we dichotomized the surgeon and AI ratings into high (ASA class III or IV) and low (ASA class I or II) categories and measured concordance and interrater agreement. Concordance as defined above and interrater agreement as measured by Cohen κ may diverge, for instance, when a large proportion of concordant cases occur by chance because the latter is designed to correct for chance agreements. This is more likely to occur with limited category sizes, such as with dichotomization of ASA class.

We used binomial multivariable logistic regression models to evaluate the association between concordance of the surgeon-rated and AI-rated ASA class and patient age, sex, time (normalized to the date of the first included surgical episode, that is, March 2024, to adjust for potential improvements in surgeon ratings over time), and presence of a prior anesthesia-rated ASA class while adjusting for the surgeon as a fixed effect. The presence of a prior anesthesia-rated ASA class was included because when this information was available to surgeons, it could have been used as an anchor for their rating, and prior data have shown that anesthesia-rated ASA class is generally concordant with that of AI models.^14^ Statistical significance was defined as *P* < .05.

### Sample Size Estimation

We assumed a distribution of ASA classification in our outpatient hand surgery sample similar to that previously described,^10^ namely, 23% ASA-I, 57% ASA-II, 19% ASA-III, and 1% ASA-IV. We calculated that at least 235 patients were needed to obtain a 95% confidence interval for an arbitrary κ estimate of 0.5 that was no wider than 0.4 to 0.6.^16^ Anticipating that some collected data may ultimately be unusable due to missingness, we aimed to collect 10% more than the required number of patients.

## Results

**Figure 1.**
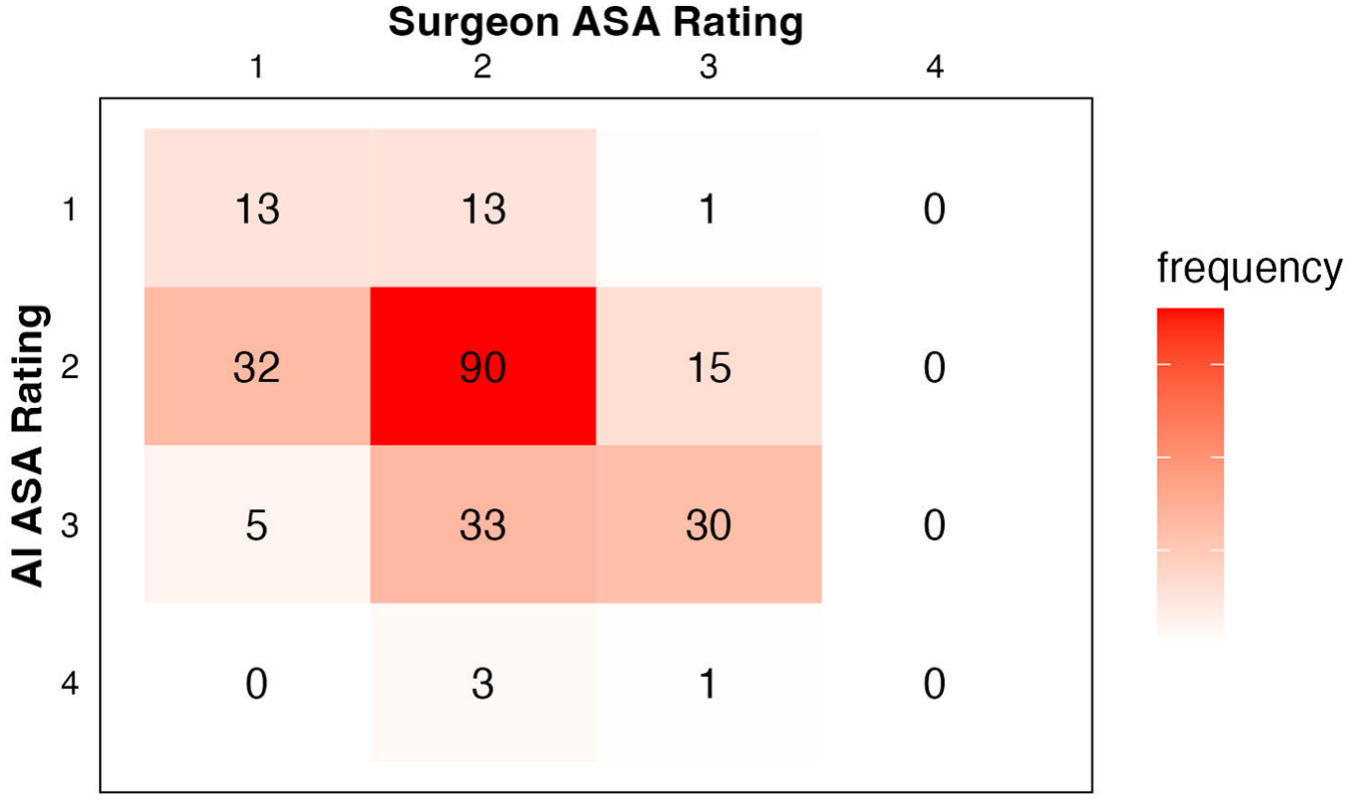
Cross-tabulated ASA classes assigned by the surgeon or ChatGPT. *Note*. ASA = American Society of Anesthesiologists; AI = artificial intelligence.

Cross-tabulated ASA classifications as rated by the surgeon and by AI are shown in Figure 1. Overall concordance between surgeon and AI ratings was 56% (133/236). Of cases where surgeon and AI ratings were discordant (n = 103), surgeon-rated ASA class was lower than the AI rating 72% of the time. Surgeon-rated ASA class exhibited fair agreement with the AI rating (κ = 0.38, *P* < .001) as well as with prior anesthesia-rated ASA class (κ = 0.36, *P* < .001). The AI-rated ASA class exhibited moderate agreement with prior anesthesia-rated ASA class (κ = 0.44, *P* < .001).

When the surgeon and AI ratings were dichotomized into high and low categories, concordance improved to 76%, but interrater agreement was still only fair (κ = 0.37, *P* < .001). In the sensitivity analysis, using the higher AI-rated ASA class (when present) reduced the concordance and agreement between surgeon and AI ratings (38% [90/236]; κ = 0.24, *P* < .001). In the sensitivity analysis, agreement between the AI and prior anesthesia rating was also lower (κ = 0.34). Therefore, only the lower AI-rated ASA class was used for all analyses. After adjusting for surgeon as a fixed effect, age, sex, and the presence of a prior anesthesia-rated ASA class were not associated with the odds of concordance between surgeon-rated and AI-rated ASA class, while slight improvements over time were noted (Table 2).

**Table 2.** Predictors of Concordance Between Surgeon and AI Rating of ASA Class.

Predictor	Adjusted odds ratio (95% confidence interval)	*P* value
Age	1.01 (0.99-1.03)	.44
Sex		
Female	Ref.	
Male	1.50 (0.87-2.62)	.15
Days since start of study	1.004 (1.0009-1.008)	.01
Prior ASA classification		
Yes	1.61 (0.89-2.91)	.11

*Note*. Adjusted for surgeon as a fixed effect. AI = artificial intelligence; ASA = American Society of Anesthesiologists.

In the entire cohort, 7 patients (3%) had an ED visit within 30 days after surgery, of which 3 (1%) were hospitalized. None of the reasons for ED visits or hospitalization were directly related to the hand surgery, although 1 patient developed wrist gout in the operative extremity after carpal tunnel release. The surgeon-rated ASA class was lower than the AI-rated ASA class in 57% (4/7) of the patients with postoperative ED visits or hospitalizations.

## Discussion

In this study, we found that surgeon-rated and AI-rated ASA class exhibited fair agreement, but surgeons tended to underestimate ASA class relative to the AI model in discordant cases. Therefore, our results suggest that generative AI could be used as a tool in assigning ASA class for hand surgeries performed under local anesthesia where an anesthesia provider is not present, with further modifications made with additional clinical information. Although the current AI model cannot yet replace human ratings of ASA class, its potential use as an assistive tool warrants further study in real-world settings.

Our results should be viewed in context with a previous study that used ChatGPT to assess ASA class in patients undergoing a wide range of general surgical procedures, which found high agreement between the AI output and anesthesiologist ratings with a κ of 0.86.^14^ In contrast, surgeon ratings showed only fair agreement with the AI rating in our study, suggesting that a standardized, AI-assisted method for assigning ASA class may both improve the accuracy of assignments and reduce surgeon burden. Indeed, we found higher agreement between the AI and prior anesthesia rating in this study. This interpretation of results is consistent with prior data showing that surgeon ASA class ratings are discordant with anesthesiologist ASA class ratings,^12^ implying that the use of AI could help more closely align the surgeon rating with that of a trained anesthesia provider.

However, further study on AI model inputs is warranted. The prior AI model that showed high agreement with anesthesiologist ratings also included more detailed patient information in the predictive model, such as medications, abnormal laboratory and imaging results, and consultation notes,^14^ which may not be directly comparable with the model used in this study. The AI model used in this study included only the patient’s past medical history, which was readily available in the EMR. This methodological choice was made to simulate a fast-paced surgical clinical setting, enabling rapid real-world clinical implementation without intensive resource or time investment. Based on our data, we advocate for surgeons using the AI output as a starting point for local anesthetic cases in ambulatory patients, with further fine-tuning of the ASA class based on the patient’s global presentation. Although the provision of more granular clinical information (eg, weight, height, smoking history, abnormal laboratory and imaging results) to AI models could further improve the accuracy of ASA ratings,^14^ this should be balanced against the time required to collect the granular clinical data and the time constraints of a busy surgical clinic.

Interestingly, surgeons tended to underestimate the ASA class compared with the AI model, despite having access to more clinical information. Similar phenomena have previously been observed. In a study in which hypothetical case vignettes were given to anesthesia and nonanesthesia providers, high discordance between nonanesthesia and anesthesia providers was noted, and surgical providers consistently underestimated the ASA class.^4^ Similarly, in another study of more than 46 000 elective surgical procedures, there was a 33% discordance rate between the surgeon and anesthesiologist ASA class ratings, with surgeons underestimating the ASA class in 79% of those cases.^12^ Interestingly, the risk of a negative clinical outcome increased when surgeons underestimated the ASA class.^12^ Because patients with ASA class IV are not typically candidates for having their surgery performed at ambulatory surgical centers due to safety concerns,^17^ underestimation of the ASA class may lead to an unintended increase in the risk profile of the ambulatory surgical facility patient population. Given that ASA class is used to determine suitability for hand surgery in the ambulatory setting, it is also likely that additional noise in the surgeon ASA ratings was introduced by the fact that little difference exists between ASA II and III, giving surgeons little incentive to accurately distinguish these, as opposed to ASA class IV in which case surgery may not be able to be performed in a freestanding ambulatory facility. Therefore, an AI-assisted strategy for ASA class assignment could assist hand surgeons in more carefully assessing higher risk patients, providing an anchor point and nudge for appropriately higher ratings.

Nevertheless, the overall utility of the ASA classification in local anesthesia–only hand surgery is unclear, especially given the low incidence of postoperative ED visits or hospitalizations in our study. As none of the ED visits were a result of having surgery under local anesthesia in the ambulatory setting, it is unlikely that they would have been prevented by altering the ASA class rating or changing to monitored anesthesia care. Hand procedures performed in the ambulatory surgical setting are generally low risk, with an adverse event rate of 0.2% in 1 large study.^17^ Given the low incidence of adverse events, the positive predictive value of a high ASA class is low for patients undergoing ambulatory hand surgery, thus potentially limiting its application. Furthermore, most patients undergoing ambulatory hand surgery have an ASA class of I or II.^17^ In a prior study, when the discordance between the surgeon and anesthesiologist on ASA class was between I and II, there was no difference in clinical outcomes; there was only a difference in clinical outcomes when the discordance was between ASA classes III and IV.^12^ Thus, given the lower overall ASA class of patients undergoing ambulatory hand surgery, the utility of assigning ASA class for hand procedures performed under local anesthesia only in the first place is questionable. Furthermore, the performance of these procedures under local anesthesia only results in the avoidance of anesthesia-related risks even in patients with higher ASA classes.

Our study contains inherent limitations. Because no anesthesia provider is present for hand procedures performed under local anesthesia, there was no gold standard ASA class rating for comparison. Instead, we compared the surgeon and AI ratings with the most recent anesthesia-rated ASA class, when present; however, the patient’s risk profile may have changed since the last procedure. Future comparative studies among hand surgeons, AI, and anesthesiologists would help further evaluate these approaches for assessing ASA in hand cases that are suitable for wide-awake surgery. Although the AI model produced ASA class assignments that were in fair agreement with the surgeon rating, further research is needed to directly compare the AI assignments with those performed by an anesthesiologist in real time. Thus, any AI-generated ASA class outputs should only be used as a clinical assistance tool. Artificial intelligence models are iterative and improve over time as the information base available to them grows. Therefore, our results should be viewed as a snapshot in time; future AI models may achieve greater accuracy. We recognize that although machine learning models could be trained to improve AI model–based ASA class assignments, we did not perform such training to make potential clinical implementation as feasible and least time-intensive/resource-intensive as possible in a fast-paced surgical environment. Although we did not record the time required to input each patient’s past medical history into the AI interface, this time was minimal, as the list was directly copied from the EMR without modification. Further studies are needed to compare the relative time burden of this approach with a formal surgeon or anesthesiologist assessment.

In summary, the main implication of this study is that generative AI could be used as a tool to improve surgeon ASA class assignment for local anesthesia–only hand surgeries in ambulatory patients. The usability of this tool in real-world hand surgical settings should be assessed in future studies. In addition, further research is needed to evaluate the utility of ASA class assignment for characterizing surgical risk in local anesthesia–only hand surgery.
